# Causal Associations between Functional/Structural Connectivity and Stroke: A Bidirectional Mendelian Randomization Study

**DOI:** 10.3390/biomedicines11061575

**Published:** 2023-05-29

**Authors:** Yisong Wang, Longtao Yang, Jun Liu

**Affiliations:** 1 Department of Radiology, The Second Xiangya Hospital of Central South University, Changsha 410011, China; wysdoctor@163.com (Y.W.); 228211091@csu.edu.cn (L.Y.); 2 Clinical Research Center for Medical Imaging in Hunan Province, Changsha 410011, China; 3 Department of Radiology Quality Control Center in Hunan Province, Changsha 410011, China

**Keywords:** resting-state, stroke, Mendelian randomization, functional network connectivity

## Abstract

Disruption of brain resting-state networks (RSNs) is known to be related to stroke exposure, but determining causality can be difficult in epidemiological studies. We used data on genetic variants associated with the levels of functional (FC) and structural connectivity (SC) within 7 RSNs identified from a genome-wide association study (GWAS) meta-analysis among 24,336 European ancestries. The data for stroke and its subtypes were obtained from the MEGASTROKE consortium, including up to 520,000 participants. We conducted a two-sample bidirectional Mendelian randomization (MR) study to investigate the causality relationship between FC and SC within 7 RSNs and stroke and its subtypes. The results showed that lower global mean FC and limbic network FC were associated with a higher risk of any ischemic stroke and small vessel stroke separately. Moreover, ventral attention network FC and default mode network SC have a positive causal relationship with the risk of small vessel stroke and large artery stroke, respectively. In the inverse MR analysis, any stroke and large artery stroke were causally related to dorsal attention network FC and somatomotor FC, respectively. The present study provides genetic support that levels of FC or SC within different RSNs have contrasting causal effects on stroke and its subtypes. Moreover, there is a combination of injury and compensatory physiological processes in brain RSNs following a stroke. Further studies are necessary to validate our results and explain the physiological mechanisms.

## 1. Introduction

Based on the 2019 Global Burden of Disease (GBD) study, stroke has been the second leading cause of death and a predominant contributor to increasing total disability-adjusted life years (DALYs) worldwide [1], which poses a significant burden at both individual and societal levels, especially in low- and middle-income countries. As the global burden of stroke increases, it is vital to identify potential risks and protective factors for stroke prevention. According to neuropathology, stroke can be divided into two main categories: ischemic stroke (IS) and hemorrhagic stroke, of which the former is the most frequent [2]. Common etiologic subtypes of IS consist in large artery stroke (LAS), cardioembolic stroke (CES), and small vessel stroke (SVS). Pathophysiological mechanisms are variable between these IS subtypes [3]. As a result, studies of IS as a whole may not be sufficient to assess the impact of risk factors under the influence of subtype distribution. Although the risk factors for stroke are numerous, the fact that genetic predisposition to functional and structural connectivity (FC/SC) within seven resting-state networks (RSNs), including default mode network, ventral attention network, dorsal attention network, visual network, limbic network, somatomotor network, and frontoparietal network, was associated with the risk of stroke subtypes has scarcely been studied. Studying the relationships between risk factors and specific stroke subtypes might provide information for obtaining more targeted neuro-imaging biomarkers.

It is estimated that approximately one-third of stroke survivors will undergo cognitive disorders manifesting in various cognitive domains within a few months (i.e., post-stroke cognitive impairment (PSCI)), which leads to a tremendous burden on victims, their families, and the community as a whole [3,4]. Illuminating the association between stroke and cognitive dysfunction is critical for a targeted intervention development of treatment strategies. FC and SC within RSNs are vital for cognitive performance. Genetic variants in RSN FC affect biological processes relevant to brain disorders. For example, lower default mode network FC was associated with a higher genetic risk of Alzheimer’s disease [5]. PSCI highlights the causal relationship between stroke and cognitive impairment. Brain network connections have shown dynamic changes throughout stroke [6]. Many studies have found that alterations of FC and SC within and between brain RSNs correlate with cognitive decline and recovery after a stroke exposure [7,8,9]. However, these observational studies focusing on different affected cognition behaviors following stroke are unclear about whether there are causal relationships between specific stroke subtypes with changes of FC and SC within 7 RSNs due to the existing cross-sectional design. Stroke lesions in brain regions have various effects on cognitive functions, and these impacts should be interpreted from an RSN perspective [10].

Most recently, Mendelian randomization (MR) has become an appealing method for inferring causal associations between exposures and outcomes using genetic variants as instrumental variables (IVs). MR analysis is based on three key hypotheses: (1) instrumental variables were closely correlated with exposure; (2) there were no observed associations between instrumental variables and confounders; and (3) the effects of instrumental variables on outcome occurred exclusively through exposure.

Therefore, we first performed bidirectional MR analysis to investigate whether FC and SC within 7 RSNs could predict the risk of stroke and its subtypes and validated whether stroke is causally related to FC and SC.

## 2. Materials and Methods

### 2.1. Data Sources

We used data on genetic variants associated with FC and SC from a recent GWAS meta-analysis, which included 24,336 participants. GWAS summary statistics for stroke and its subtypes were retrieved from the MEGASTROKE consortium, involving 40,585 stroke cases and 406,111 controls of European ancestry. IS cases were further subdivided into large artery stroke (LAS, *n* = 4373), cardioembolic stroke (CES, *n* = 7193), and small vessel stroke (SVS, *n* = 5386) based on the Trial of Org 10172 in Acute Stroke Treatment criteria. In order to perform bidirectional MR analysis, GWAS of the FC and SC was used as the outcome dataset.

### 2.2. Selection of Genetic Variants

We selected single nucleotide polymorphisms (SNPs) associated with FC and SC, stroke and its subtypes at the genome-wide significance threshold (*p* < 5 × 10^−6^) as candidate IVs for the following MR analysis. Then, F-statistics were calculated to test the strength of these selected SNPs. IVs are generally considered a robust instrument when F-statistics are above 10. We chose independent SNPs (r^2^ < 0.001), calculated the F-statistic, and manually excluded SNPs correlated with confounders.

### 2.3. Statistical Analysis

The inverse-variance-weighted (IVW) method was selected as a top-choice approach for determining the causal effects of exposures on outcomes. In sensitive analysis, we used other MR approaches to ensure the robustness of our results, as follows: (1) We performed MR Egger, weighted median, simple mode, and weighted mode as complementary methods to detect the stability and reliability of our results; (2) Statistical heterogeneity among SNPs was measured using Cochran’s Q test in the IVW technique (*p* < 0.05); (3) MR-Egger regression’s intercept was calculated to evaluate whether genetic variants have pleiotropy; (4) In addition, MR-pleiotropy residual sum and outlier (MR-PRESSO) global test was also employed to identify outlier SNPs and potential horizontal pleiotropy (*p* was set at 0.05). After observing heterogeneity or horizontal pleiotropy, we recalculated MR estimates after deleting the outlier SNPs detected by MR-PRESSO.

## 3. Results

### 3.1. The Causal Effects of FC and SC Levels on Stroke

Significant SNPs (*p* < 5 × 10^−6^) associated with FC and SC in RSNs were obtained (Table S1). The harmonized SNPs are displayed in Table S2. The complete results of MR analysis are shown in Table S3. In the MR analysis, two exposures were significantly associated with SVS: limbic network FC (β: −33.048, 95% CI: [−56.856, −9.240], *p* = 0.007) and ventral attention network FC (β: 122.976, 95% CI: [57.513, 188.438], *p* = 0.00023). Moreover, the IVW method indicated that genetically increased global mean FC was associated with a lower risk of AIS (β: −20.549, 95% CI: [−40.059, −1.039], *p* = 0.039), whereas increased default mode network SC indicated a higher risk of LAS (β: 37.737, 95% CI: [5.786, 69.687], *p* = 0.021). The results evaluated by other supplementary MR methods were directionally consistent with the IVW method, as shown in Table 1. Heterogeneity tests indicated no heterogeneities of the genetic variants; pleiotropy tests indicated no pleiotropy of the genetic variants except the MR estimate between global mean FC and CES, as shown in Table 2 and Table S4.

### 3.2. The Causal Effects of Stroke on FC and SC Levels

To further explore the causal effects of stroke and its subtypes on FC and SC levels, we performed a bidirectional MR analysis. Significant SNPs associated with stroke types were obtained (Table S5). A total of 47, 45, 34, 35, and 31 SNPs significantly associated with AS, AIS, LAS, CES, and SVS were harmonized (Table S6). We found that genetic predisposition to AS (β: 2.938 × 10^−4^, 95% CI: [1.420 × 10^−5^, 5.732 × 10^−4^], *p* = 0.047) and genetic predisposition to LAS (β: −5.079 × 10^−4^, 95% CI: [−1.010 × 10^−3^, −5.617 × 10^−6^], *p* = 0.039) were highly correlated with dorsal attention network FC and somatomotor network FC, respectively (Table 1 and Table S7). No heterogeneity and pleiotropy were identified in the analysis of AS (all *p* > 0.1) (Table 2 and Table S8).

## 4. Discussion

To our knowledge, this is the first study to investigate whether the strength levels of FC and SC within 7 RSNs are causally associated with the risk of stroke subtypes and to further evaluate whether there are reverse causal effects of stroke subtypes on FC and SC by using the bidirectional MR method. The results, supported by multiple statistical methods, showed that lower global mean FC and limbic network FC were associated with a higher risk of AIS and SVS, separately. Moreover, this study provides evidence for positively causal relationships of ventral attention network FC and default mode network SC with the risk of SVS and LAS, respectively. Inverse MR analysis indicated that AS and LAS were causally related to dorsal attention network FC and somatomotor FC, respectively.

In previous research, risk factors of stroke have been intensively studied [11,12], while little was known about whether there were causal effects of FC and SC levels within RSNs on stroke and its subtypes. The associations between FC and SC within RSNs and stroke were complicated. Until now, 10 modifiable factors have been correlated with an increased risk of stroke, such as high blood pressure, diabetes mellitus, hyperlipidemia, and smoking, which account for nearly 90% of the risk of stroke [13]. Growing evidence recently demonstrated an inverse causal association between education and IS [14,15,16]. Several MR studies also proved that a higher educational attainment was a protective factor for hypertension and type 2 diabetes, further supporting that high education could reduce IS risk [17,18]. Furthermore, the education level might influence stroke survivors’ cognitive status [19,20]. Higher education levels were less likely to affect their cognitive functioning because they had a larger brain reserve capacity [21]. Indeed, cognition and education are mutually related and inseparable, with solid genetic evidence supporting a bidirectional relationship between educational attainment and cognitive function from previous GWAS studies [18,22,23]. Higher educational attainment can improve cognitive performance. Maintaining cognitive performance depends on FC and SC within 7 RSNs. Higher global mean FC indicates a better cognitive function. The limbic network is commonly responsible for emotion regulation, episodic memory, and action-outcome learning, where the hippocampus is associated with learning, cognitive functions, and memory, in particular converting short-term memory into long-term memory. Given that education and cognition are strongly genetically correlated (rg = 0.662) [24], we speculate that educational attainment is somewhat reflected in the strength of global mean FC and limbic network FC. Thus, genetically predicted global and limbic network FCs were inversely associated with IS risk. Public health, educational policy and governmental decision-making should reflect the importance of education for stroke prevention.

The potential mechanism according to which higher ventral attention network FC and default mode network FC are positively associated with the risk of LAS and SVS separately might be attributed to the neuro-vascular coupling hypothesis. Blood perfusion is fine-tuned to deliver blood with a higher need [25]. Therefore, brain RSNs with high FC and SC have a higher spontaneous neuronal activity and, thus, a higher metabolic demand, resulting in more cerebral perfusion. Traditional vascular risk factors promote the progression of atherosclerotic stenosis and thrombus in atherosclerotic arteries. On this basis, elevated levels of circulating blood flow due to an increased activity of RSNs may be a potential mechanism leading to an increased risk of IS. This MR study did not identify any causal role with CES. The possible reason for this may be that most CES cases are caused by atrial fibrillation, which has different risk factors than other stroke subtypes [12].

Reverse MR analysis suggested that the occurrence of LAS was a risk factor for reduced FC strength in the somatomotor network. Previous epidemiological studies consistently reported disruptions and recovery of functional brain networks over time after a stroke exposure. Most studies ignored that there might be causal associations between IS subtypes and the strength of FC and SC within specific RSNs [8,9,26]. Extensive functional network connectivity changes occur immediately after stroke and have important prognostic implications [27]. Post-stroke changes in brain networks may be a combination of neurological damage and compensation. Actually, the impaired cognitive domains after stroke might vary depending on the stroke characteristics, such as stroke subtypes, volume, number, location, and severity [28,29]. Attention, working memory, and executive function are most commonly affected [20,30]. Prior RSN FC research demonstrated that the functional connectivity between the ipsilesional and the contralesional primary sensorimotor cortex was significantly impaired at the early stage of stroke [31,32]. Functional connections cannot be separated from integrated structural connections. Following a stroke, patients’ brains will suffer structural damage due to ischemia or hemorrhage [33,34]. The underlying mechanisms of neurological deficits following stroke are complex. For instance, ischemic injury will result in a sequence of cascade reactions involving hypoperfusion, calcium overload, oxidative stress, blood-brain barrier dysfunction, and neuroinflammation responses. From a pathological perspective, stroke damages structural and functional synaptic plasticity, impairing synaptic connections’ strength and the efficiency of synaptic transmission [35,36]. These findings shed light on neuro-mechanisms from a network-level prospective, which may contribute to explaining why patients after stroke exposure show deficits of behavior and cognition.

This study also found that a higher genetic risk of AIS was associated with higher dorsal attention network FC. Increased functional connectivity in the dorsal attention network might represent a compensatory effect, which contributed to maintaining normal cognitive performance in the early stages of stroke. In this study, the findings did not indicate any causal role between stroke subtypes and other RSNs. One possible reason was that primary motor- and vision-related functions depend on specific RSNs. In contrast, advanced cognitive functions, such as attention and memory, rely more on the communication between several RSNs than on the function of any individual RSN [37].

This MR study has several strengths. First, this study effectively avoided the disadvantages of traditional observational research methods, such as uncertain confounding factors and reverse causality. Second, our summary-level data were derived from the latest GWAS of populations with European ancestry, which increased our statistical power and provided compelling evidence of associations. Moreover, we used some alternative approaches to estimate possible pleiotropic bias and attained consistent effect estimates, which confirmed the robustness of our findings. Finally, we analyzed the relationship between FC and SC within 7 RSNs and stroke and its subtypes for the first time, which, to some extent, had yet to be investigated in previous studies.

Despite these advantages, one should be aware of some limitations in this study. First, this analysis was primarily based on populations of European ancestry, restricting the generalizability to other people. Second, in this study, the β value might not represent the severity of influence, merely indicating that there was a causal relationship. Third, there was significant pleiotropy in the MR estimate between global mean FC and CES, even though the MR result was significant. Finally, our study focused exclusively on abnormalities of FC and SC within a single RSN associated with stroke and its subtypes. Brain functions, such as language, motor, and primary sensory functions, are relatively confined to specific brain networks, whereas high-level cortical functions, such as memory and executive functions, appear to depend on communication between several brain RSNs. In addition to causing damage at the location of the injury, stroke also affects long-distance anatomical connections and disrupts the functional link between different brain RSNs. Moreover, though our reverse MR analysis results were not significant enough, they could be suggestive. Further investigations with representative samples will be required to investigate causal relationships between cerebral connectivity and stroke as well as its subtypes.

## 5. Conclusions

This study provides genetic evidence that the levels of FC and SC within different RSNs have opposite causal effects on stroke and its subtypes. Moreover, there is a combination of injury and compensatory physiological processes in brain RSNs following a stroke. Further clinical trials are warranted to investigate the direct causal association of FC and SC within brain RSNs and stroke and its subtypes.

## Figures and Tables

**Table 1:** Full positive results of MR estimate for the association between functional/structural connectivity and strokes.

Functional/Structural Connectivity (Exposure)	Stroke Type (Outcome)	MR Method	No. SNP	F	β (95% CI)	*p*-Value
Global mean FC	AIS	IVW(MRE)	8	25.52	−20.549 (−40.059, −1.039)	0.03899
		MR Egger	8		−59.964 (−109.570, −10.357)	
		Weighted median	8		−24.254 (−55.788, 7.279)	
		Simple mode	8		−28.694 (−76.351, 18.964)	
		Weighted mode	8		−28.132 (77.790, 21.525)	
Limbic network FC	SVS	IVW (MRE)	10	22.63	−33.048 (−56.856, −9.240)	0.00652
		MR Egger	10		−22.480 (−164.960, 120.000)	
		Weighted median	10		−43.583 (−89.394, 2.229)	
		Simple mode	10		−53.689 (−126.947, 19.568)	
		Weighted mode	10		−50.592 (−114.007, 12.823)	
Ventral attention network FC	SVS	IVW (MRE)	4	21.63	122.976 (57.513, 188.438)	0.00023
		MR Egger	4		129.368 (−145.057, 403.792)	
		Weighted median	4		103.820 (−33.168, 240.808)	
		Simple mode	4		66.841 (−107.596, 241.278)	
		Weighted mode	4		171.079 (−6.787, 348.945)	
Default mode network SC	LAS	IVW (MRE)	14	21.06	37.737 (5.786, 69.687)	0.02062
		MR Egger	14		85.152 (−2.216, 172.521)	
		Weighted median	14		53.524 (5.341, 101.706)	
		Simple mode	14		67.920 (−15.131, 150.971)	
		Weighted mode	14		66.506 (−6.566, 139.577)	
/						
**Stroke Type (Exposure)**	**Functional/Structural Connectivity (Outcome)**	**MR Method**	**No. SNP**	**F**	**β (95% CI)**	***p*** **-Value**
AS	Dorsal attention network FC	IVW (MRE)	47	48.41	2.938 × 10^−4^ (1.420 × 10^−5^, 5.732 × 10^−4^)	0.03946
		MR Egger	47		6.562 × 10^−4^ (−2.428 × 10^−4^, 1.555 × 10^−3^)	
		Weighted median	47		2.888 × 10^−4^ (1.086 × 10^−4^, 6.862 × 10^−4^)	
		Simple mode	47		3.669 × 10^−4^ (−5.547 × 10^−4^, 1.288 × 10^−3^)	
		Weighted mode	47		3.669 × 10^−4^ (5.267 × 10^−4^, 1.261 × 10^−3^)	
LAS	Somatomotor network FC	IVW (MRE)	34	29.97	−5.079 × 10^−4^ (−1.010 × 10^−3^, −5.617 × 10^−6^)	0.04749
		MR Egger	34		−2.450 × 10^−6^ (−1.420 × 10^−3^, 1.414 × 10^−3^)	
		Weighted median	34		−5.920 × 10^−4^ (−1.538 × 10^−3^, 3.539 × 10^−4^)	
		Simple mode	34		−5.828 × 10^−4^ (−2.644 × 10^−3^, 1.478 × 10^−3^)	
		Weighted mode	34		−6.024 × 10^−4^ (−2.333 × 10^−3^, 1.129 × 10^−3^)	

IVW: inverse variance weighted; MRE: multiplicative random effects model; MR Egger: Mendelian randomization-Egger; OR: odds ratio; CI: confidence interval; No. SNP: number of single nucleotide polymorphisms; AS: any stroke; AIS: any ischemic stroke; LAS: large artery stroke; CES: cardioembolic stroke; SVS: small vessel stroke; FC: functional connectivity; SC: structural connectivity.

**Table 2:** Sensitivity analyses for association between functional/structural connectivity and strokes.

Functional/Structural Connectivity (Exposure)	Stroke Type (Outcome)	No. SNP	Pleiotropy		Heterogeneity	
			MR-PRESSO Global *p*-Value	MR-Egger *p*-Value	IVW Test *p*-Value	MR-Egger *p*-Value
Global mean FC	AIS	8	0.7266	0.1244	0.9700	0.7185
Limbic network FC	SVS	10	0.9065	0.8845	0.9010	0.8451
Ventral attention network FC	SVS	4	0.7768	0.9648	0.7821	0.5837
Default mode net-work SC	LAS	14	0.7342	0.2649	0.6894	0.7293
/						
**Stroke Type (Exposure)**	**Functional/Structural Connectivity (Outcome)**	**No. SNP**	**Pleiotropy**		**Heterogeneity**	
			**MR-PRESSO Global *p*-Value**	**MR-Egger *p*-Value**	**IVW Test *p*-Value**	**MR-Egger *p*-Value**
AS	Dorsal attention network FC	47	0.4479	0.4099	0.4484	0.4357
LAS	Somatomotor network FC	34	0.9920	0.4279	0.9894	0.9890

MR-PRESSO: Mendelian Randomization Pleiotropy RESidual Sum and Outlier; MR-egger: Mendelian randomization-Egger; IVW: inverse variance weighted; No. SNP: number of single nucleotide polymorphisms; AIS: any ischemic stroke; SVS: small vessel stroke; LAS: large artery stroke; AS: any stroke; FC: functional connectivity; SC: structural connectivity.

## Data Availability

Functional/structural connectivity within RSNs data from https://www.fmrib.ox.ac.uk/ukbiobank/gwas_resources/index.html and stroke data from https://megastroke.org are both open access.

## Supplementary Materials

The following supporting information can be downloaded at: https://www.mdpi.com/article/10.3390/biomedicines11061575/s1, Table S1: GWAS summary data of functional/structural connectivity at *p*-value < 5.0 × 10^−6^; Table S2: Detailed information of instrumental variables used in MR analyses; Table S3: Full result of MR estimates for the association between functional/structural connectivity and stroke types; Table S4: Sensitivity analysis of instrumental variables used in MR analyses; Table S5: GWAS summary data of stroke types at *p*-value < 5.0 × 10^−6^; Table S6: Detailed information of instrumental variables used in inverse MR analyses; Table S7: Full result of inverse MR estimates for the association between functional/structural connectivity and stroke types; Table S8: Sensitivity analysis of instrumental variables used in inverse MR analyses.
